# Efficacy and safety of concomitant use of proton pump inhibitors with aspirin-clopidogrel dual antiplatelet therapy in coronary heart disease: A systematic review and meta-analysis

**DOI:** 10.3389/fphar.2022.1021584

**Published:** 2023-01-10

**Authors:** Xiaofeng Luo, Min Hou, Shuangshuang He, Xue Yang, Pan Zhang, Yingxin Zhao, Haiyan Xing

**Affiliations:** Department of Pharmacy, Daping Hospital, Army Medical University, Chongqing, China

**Keywords:** proton pump inhibitors, aspirin, clopidogrel, coronary heart disease, medication interaction, meta-analysis

## Abstract

**Background:** Proton pump inhibitors (PPIs) are usually prescribed to prevent gastrointestinal (GI) complications in patients receiving dual antiplatelet therapy (DAPT). This systematic review and meta-analysis aimed to explore the efficacy and safety of the concomitant use of PPIs with aspirin-clopidogrel DAPT in patients with Coronary heart disease (CHD).

**Method:** The PubMed, Embase, Cochrane Library, and Web of Science databases were searched from inception to August 2022 for eligible studies. The adjusted hazard ratios (HRs) and 95% confidence intervals (CIs) were calculated to evaluate the clinical outcomes. Subgroup analysis was conducted according to different PPI subtypes, populations, follow-up times and study types. This study was registered on PROSPERO (CRD42022332195).

**Results:** A total of 173,508 patients from 18 studies [2 randomized controlled trials (RCTs), 3 *post hoc* analyses of RCTs, and 13 cohort studies] were included in this study. Pooled data revealed that coadministration of PPIs significantly increased the risk of major adverse cardiovascular events (MACEs) (HR = 1.15, 95% CI = 1.06–1.26, *p* = .001) and reduced the risk of gastrointestinal (GI) complications (HR = 0.44, 95% CI = 0.30–0.64, *p <* .0001). Subgroup analysis results showed that the esomeprazole users and patients with coronary stenting in the PPI group were associated with an increased risk of MACEs compared with the non-PPI group. The occurrence of MACEs in PPI users was more common than that in non-PPI users in long-term follow-up (≥12 months) studies and in the observational studies. There was no significant differences in the incidences of net clinical adverse events (NACEs), all-cause mortality, or cardiac death between the two groups.

**Conclusion:** In patients with CHD, the concomitant use of PPIs with aspirin and clopidogrel was associated with a reduced risk of GI complications but could increase the rates of MACEs (particularly in patients receiving esomeprazole or with coronary stenting). There was no clear evidence of an association between PPI use and NACEs, all-cause mortality, or cardiac death. The results could have been affected by the follow-up time and study type. Further large-scale RCTs with long-term follow-up are needed.

## 1 Introduction

Coronary heart disease (CHD) is one of the most common chronic illnesses and is the leading cause of death worldwide (Zhou et al., 2019; Voutilainen, et al., 2022). Dual antiplatelet therapy (DAPT) with aspirin plus clopidogrel is recommended for patients with CHD to reduce the risk of ischemic cardiovascular events while increasing the risk of bleeding compared with either of the regimens alone (Diener et al., 2004; Benavente et al., 2012; Valgimigli et al., 2018). Gastrointestinal (GI) bleeding accounts for a significant proportion of bleeding complications in DAPT, which can lead to DAPT cessation and has been identified as an independent risk factor for poor prognosis. (Capodanno et al., 2018). Because aspirin damages the gastric mucosa by suppressing the synthesis of prostaglandins (PGs) (Nishida et al., 2011), the antiangiogenic effects of clopidogrel could impair the healing of gastric erosions (Luo et al., 2016). These patients are frequently prescribed proton pump inhibitors (PPIs) to minimize GI complications (involving ulcers and bleeding) (Levine et al., 2016; Valgimigli et al., 2018). However, previous studies have indicated that coadministration of PPIs with aspirin-clopidogrel DAPT could be associated with adverse drug-drug interactions.

Aspirin is mainly absorbed in the acidic environment. PPIs inhibit gastric acid production and increase gastric pH, resulting in poor aspirin absorption (Gesheff et al., 2014; Vaduganathan and Bhatt, 2016). Clopidogrel is a prodrug that depends on cytochrome P450 isoenzyme (mainly CYP2C19) to metabolize into an active form. PPIs are also metabolized by CYP enzymes and thus could inhibit the conversion of clopidogrel into its active metabolite (Furuta et al., 2017). Furthermore, mounting clinical data have shown that long-term intake of PPIs increases the susceptibility of patients to serious adverse events, including cardiovascular events and damage to the lower GI tract (Lue and Lanas, 2016; Xie et al., 2019; Marlicz et al., 2022; Zhai et al., 2022). However, the existing clinical studies of the association between cardiovascular events and the concomitant use of PPIs with aspirin-clopidogrel DAPT in CHD have been conflicting (Ben Ghezala et al., 2022). Some meta-analyses were conducted to assess the clinical significance of this interaction and found that coadministration of PPIs could increase the rates of major adverse cardiovascular events (MACEs), stroke, revascularization, and stent thrombosis (ST) but not myocardial infarction (MI), all-cause mortality, or cardiac death (Guo et al., 2021; Hu et al., 2018; Li et al., 2021; Melloni et al., 2015; Sherwood et al., 2015). Interestingly, two recent clinical studies reported that the rates of MI, all-cause mortality and cardiac death were significantly increased in PPI users compared to non-users (Maret-Ouda et al., 2021; Mohammed et al., 2021). In these studies, hazard ratios (HRs) containing the status of event occurrence and the time when events happened were calculated by multivariable Cox proportional hazards regression models (Spruance et al., 2004). Moreover, net clinical adverse events (NACEs) are also an important clinical outcome in CHD (Chandrasekhar et al., 2017). However, to the best of our knowledge, no meta-analyses of previous studies have reported NACEs outcomes. In addition, the length of follow-up is vital for the clinical outcome evaluation of PPI coadministration, but it has rarely been considered in previous meta-analyses. Furthermore, the results of clinical studies have also been inconsistent regarding the protective effects of PPIs in the GI tract.

Therefore, we performed this meta-analysis to evaluate the efficacy and safety of the combination treatment of PPIs with aspirin-clopidogrel DAPT for CHD patients by extracting adjusted HRs to provide a theoretical basis for clinical, individualized practice. Furthermore, subgroup analysis was conducted according to different PPI subtypes, populations, follow-up times and study types to analyze the heterogeneity.

## 2 Methods

### 2.1 Search strategy

This study was conducted in adherence to the Preferred Reporting Items for Systematic Reviews and Meta-analyses (PRISMA) guidelines. The study was registered on PROSPERO (CRD42022332195). We searched the PubMed, Embase, the Cochrane Library, and Web of Science databases for relevant studies published in English from inception to August 2022. The following Medical Subject Headings (MeSH) and keywords were used for the literature retrieval: “proton pump inhibitors (PPIs),” “dual antiplatelet therapy (DAPT),” “aspirin,” “clopidogrel,” “acute coronary syndrome (ACS),” “myocardial infarction (MI),” “percutaneous coronary intervention (PCI),” and “coronary stenting.” We also searched conference abstracts and reviewed reference lists of relevant review articles to provide additional citations.

### 2.2 Study selection

Studies were selected based on the following inclusion criteria: 1) subjects: patients with ACS, PCI, or coronary stenting receiving aspirin-clopidogrel DAPT; 2) exposure intervention: the experimental group was treated with PPIs, whereas the control group was treated with a placebo or no PPIs; 3) outcome measures: the primary outcome was MACEs, and the secondary outcomes were NACEs, MI, stroke, revascularization, ST, all-cause mortality, cardiac death, and GI complications (involving ulcers and bleeding events); and 4) study design: randomized, controlled trials (RCTs) and observational studies.

Studies were excluded if they met the following exclusion criteria: 1) patients receiving aspirin or clopidogrel alone; 2) control group patients receiving H_2_ receptor antagonists; 3) effect estimates of adjusted HRs and corresponding 95% confidence intervals (CIs) was not provided; 4) different reports of the same trial or duplicate data; and 5) case reports.

### 2.3 Data extraction and quality assessment

Two reviewers independently extracted data from eligible studies, including authors, publication year, region, population, type of PPI, follow-up time, study endpoints, study design, and sample size. The Jadad Scale and Newcastle-Ottawa Scale (NOS) scoring methods were used to assess the quality of RCTs and observational studies, respectively. Post hoc analyses of RCTs were regarded as observational studies to evaluate study quality. Discrepancies in data extraction and quality assessment, if necessary, were resolved by consultation with a third reviewer.

### 2.4 Statistical analysis

Review Manager software, version 5.3, was used for the analysis of adjusted HRs. Between-study heterogeneity was calculated with Higgins’s *I* ^*2*^ test: *I* ^*2*^ > 50% could represent substantial heterogeneity, and a random-effect model was applied; otherwise, a fixed-effect model was used. Subgroup analysis was conducted based on the PPI subclass, follow-up time, population, and study type. We estimated publication bias through a visual inspection of funnel plots.

## 3 Results

### 3.1 Search results and quality evaluation

**FIGURE 1:**
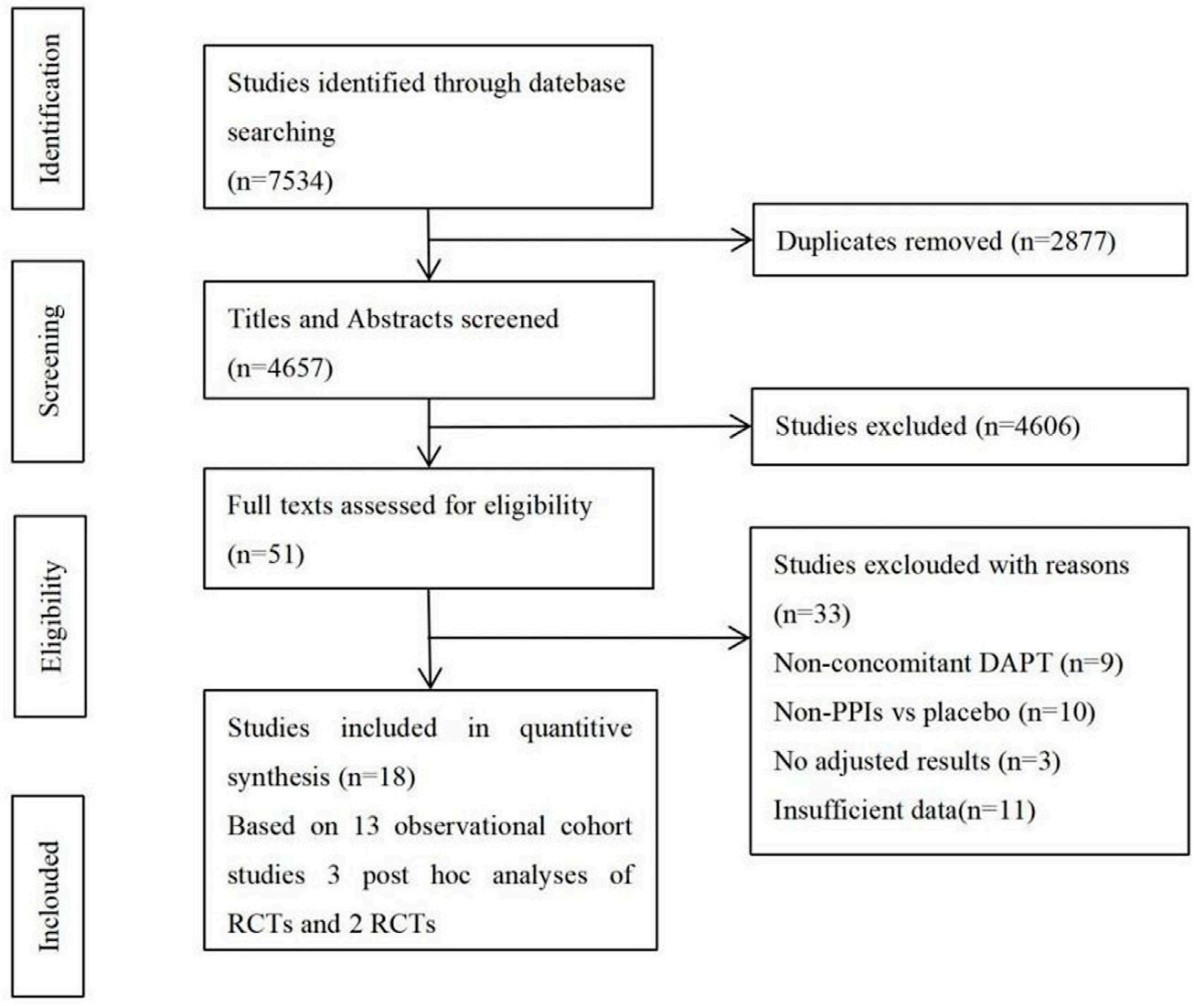
Flowchart of study selection.

The flowchart of study selection is shown in Figure 1. There were 7,534 studies identified in the preliminary electronic database search. After tiered screening, a total of 18 articles were selected for the quantitative analysis, including 2 RCTs (Bhatt et al., 2010; Gargiulo et al., 2016), 3 *post hoc* analyses of RCTs (Goodman, 2012; O'Donoghue, 2009; Simon, 2011), and 13 cohort studies (Aihara, 2012; Burkard, 2012; Chandrasekhar, 2017; Gaglia, 2010; Harjai, 2011; Hokimoto, 2014; Maret-Ouda, 2021; Mohammed, 2021; Sarafoff, 2010; Tentzeris, 2010; Weisz, 2015; Zhu, 2017; Zou, 2014). PPIs were used by 64,784 of the 173,508 patients (37.34%), and 108,700 patients did not use PPIs. Table 1 presents the main characteristics of the included studies, and the results of the quality evaluation are listed in Supplementary Tables S1, S2.

**TABLE 1:** Main characteristics of included studies.

Author, year	Country	Population	Study design	Sample (PPIs/No PPIs)	Fellow-up (months)	PPIs
Mohammed et al. (2021)	Egypt	PCI	Cohort	375/175	18	NR
Maret-Ouda et al. (2021)	Swedish	PCI	Cohort	35772/64064	12	O, E, P
Zhu et al. (2017)	China	PCI	Cohort	2142/5725	24	NR
Chandrasekhar et al. (2017)	Fifteen centers from US and Europe	CS	Cohort	1062/3573	24	NR
Gargiulo et al. (2016)	Italy	CS	RCT	738/1232	24	O, E, P, R
Weisz et al. (2015)	US and Germany	CS	Cohort	2697/5885	24	NR
Zou et al. (2014)	China	CS	Post hoc	6188/1465	12	O, E, P
Hokimoto et al. (2014)	Japan	CS	Cohort	50/124	18	R
Goodman et al. (2012)	Europe,US,Asia	ACS	Post hoc	6539/12062	12	O, E, P, L, R
	Middle East, Africa, Australia					
Aihara et al. (2012)	Japan	CS	Cohort	1068/819	12	O, E, L
Simon et al. (2011)	France	MI	Post hoc	1453/900	12	O
Harjai et al. (2011)	United States of America	PCI	Cohort	751/1900	6	O
Burkard et al. (2012)	Switzerland	CS	Cohort	109/692	36	R
Bhatt et al. (2010)	393 sites in 15 countries	ACS or CS	RCT	1876/1885	15	O, P
Gaglia et al. (2010)	United States of America	CS	Cohort	318/502	12	O, E, P, L, R
Tentzeris et al. (2010)	Austria	CS	Cohort	691/519	12	O, E, P, L, R
Sarafoff et al. (2010)	Germany	CS	Cohort	698/2640	1	O, E, P, L, R
O'Donoghue et al. (2009)	United States of America and Europe	ACS with PCI	Post hoc	2257/4538	6	O, E, P, L

*NR*, not reported; *O*, omeprazole; *E*, esomeprazole; *P,* pantoprazole; *R*, rabeprazole; *L*, lansoprazole; *RCT*, randomized controlled trial; *CS*, coronary stenting; *US*, the Unite States.

### 3.2 Quantitative synthesis

#### 3.2.1 The primary outcome

**FIGURE 2:**
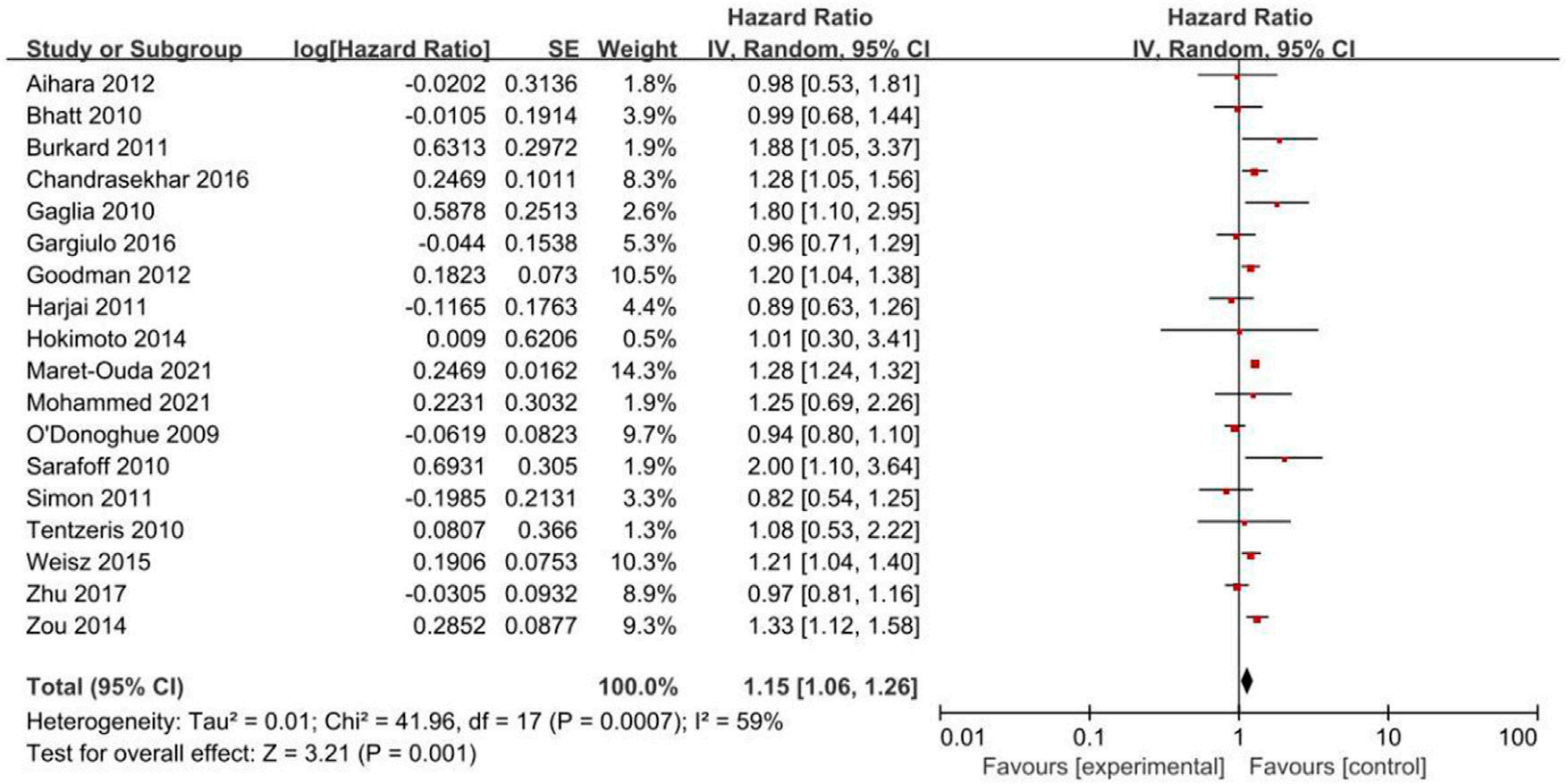
Forest plots of the risk of MACEs.

Eighteen studies reported MACEs (Figure 2). The results indicated that PPIs significantly increased the occurrence of MACEs (HR = 1.15, 95% CI = 1.06–1.26; *p* = .001) with a random-effect model (P = .0007, *I* ^*2*^ = 59%).

Subgroup analyses of PPI subclasses, populations, follow-up times and study types were performed (Table 2).

**TABLE 2:** Subgroup analysis of MACEs.

Outcome	Subgroup		Number of studies	Pooled HR (95%CI)	*P*-value	Heterogeneity		Analysis model
						*P*_h_	*I* ^*2*^	
MACEs	Type of PPIs	Omeprazole	4	1.02 (0.82–1.26)	0.87	0.03	67	R
		Esomeprazole	3	1.23 (1.06–1.42)	0.006	0.54	0	F
		Pantoprazole	3	1.25 (0.90–1.74)	0.19	0.01	77	R
		Lansoprazole	2	0.96 (0.61–1.50)	0.85	0.40	0%	F
	Study design	RCTs	2	0.97 (0.77–1.23)	0.80	0.89	0%	F
		Observational studies	16	1.17 (1.07–1.28)	0.0005	0.001	60%	R
	Population	Coronary stenting	10	1.26 (1.16–1.38)	<0.00001	0.28	18%	F
		Mixed	8	1.05 (0.92–1.21)	0.46	<0.0001	77%	R
	Follow-up time	<12 months	5	0.98 (0.86–1.11)	0.73	0.19	34%	F
		≥12 months	13	1.26 (1.23–1.30)	<0.00001	0.04	45%	R

With regard to the types of PPIs, the use of esomeprazole (HR = 1.23, 95% CI = 1.06–1.42; *p* = .006) was associated with a significant increase in the risk of MACEs but not omeprazole (HR = 1.02, 95% CI = 0.82–1.26; *p* = .87), pantoprazole (HR = 1.25, 95% CI = 0.90–1.74; *p* = .19), or lansoprazole (HR = 0.96, 95% CI = 0.61–1.50; *p* = .85). Only one study reported MACEs in patients treated with rabeprazole; therefore, subgroup analysis was not possible for rabeprazole users.

In the stratification analyses by population, we found that the occurrence of MACEs was higher in the coronary stenting group administered PPIs (HR = 1.26, 95% CI = 1.16–1.38; *p <* .00001) but not in the mixed group (HR = 1.05, 95% CI = 0.92–1.21; *p* = .46).

When stratified by length of follow-up, there was no significant difference in the incidences of MACEs between PPI users and non-PPI users in the short-term follow-up (<12 months) group (HR = 0.98, 95% CI = 0.86–1.11; *p* = .73). However, in the long-term follow-up (≥12 months) group, the occurrence of MACEs was higher in the patients administered PPIs (HR = 1.26, 95% CI = 1.23–1.30; *p <* .00001).

Subgroup analysis of observational studies (HR = 1.17, 95% CI = 1.07–1.28; *p* = .0005) showed that PPIs increased the occurrence of MACEs, while analysis of the RCTs (HR = 0.97, 95% CI = 0.77–1.23; *p* = .80) did not demonstrate statistical significance.

#### 3.2.2 The secondary outcomes

**FIGURE 3:**
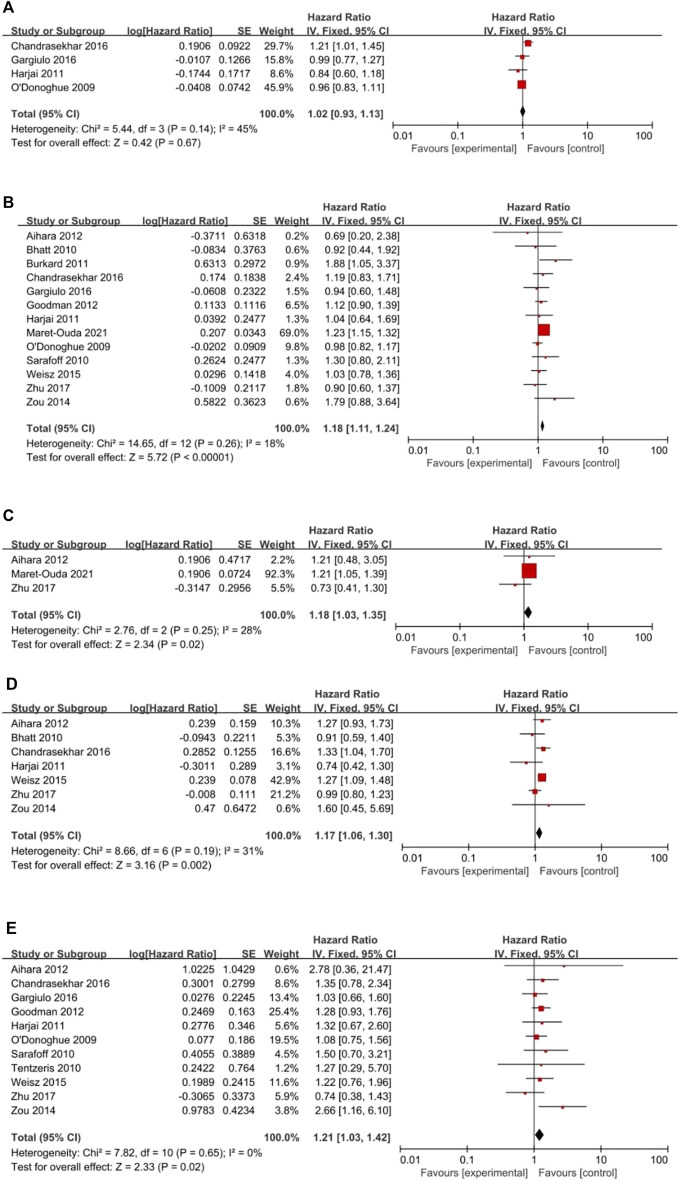
(Continued). Forest plots of **(A)** NACEs, **(B)** MI, **(C)** Stroke, **(D)** Revascularization and **(E)** Stent thrombosis.

**FIGURE 4:**
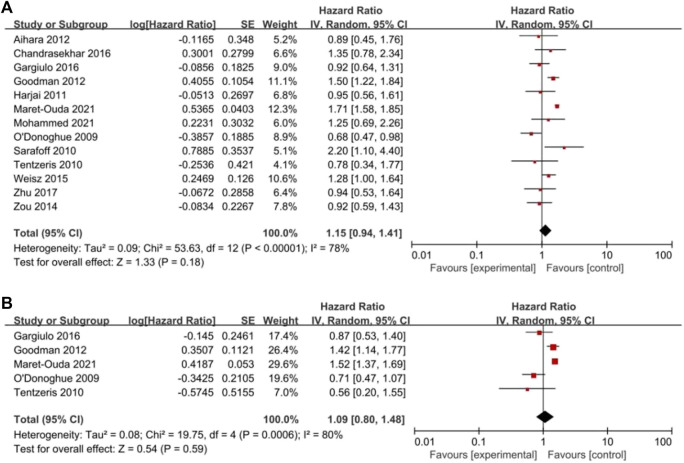
Forest plots of **(A)** all-cause mortality and **(B)** cardiac death.

**FIGURE 5:**
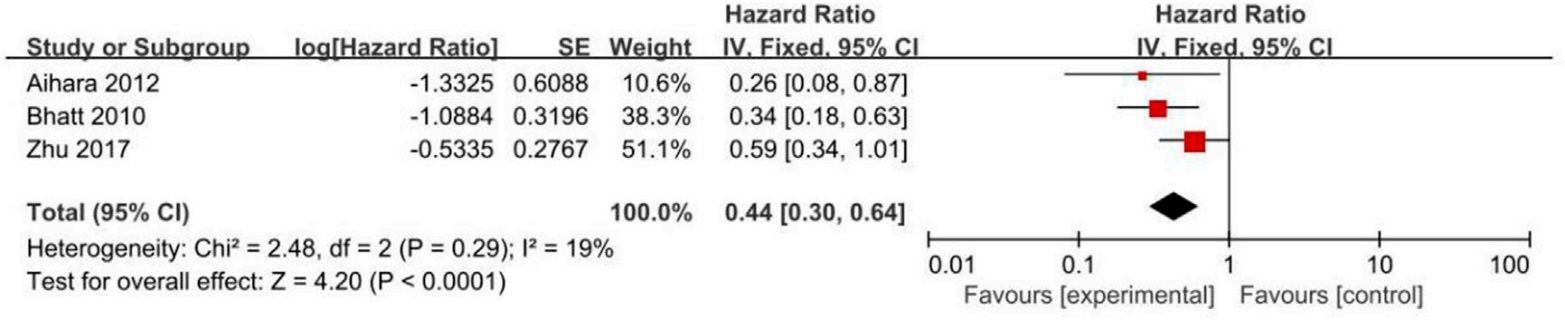
Forest plots of GI complications.

All clinical outcomes are shown in Table 3. PPIs were associated with a significant increase in the risk of MI (HR = 1.18, 95% CI = 1.11–1.24; *p <* .00001, *I* ^*2*^ = 18%, Figure 3B), stroke (HR = 1.18, 95% CI = 1.03–1.35; *p* = .02, *I* ^*2*^ = 28%, Figure 3C), revascularization (HR = 1.17, 95% CI = 1.06–1.30; *p* = .02, *I* ^*2*^ = 31%, Figure 3D), and ST (HR = 1.21, 95% CI = 1.03–1.42; *p* = .02, *I* ^*2*^ = 0%, Figure 3E) but not NACEs (HR = 1.02, 95% CI = 0.93–1.13; *p* = .67, *I* ^*2*^ = 45%, Figure 3A), all-cause mortality (HR = 1.15, 95% CI = 0.94–1.41; *p* = .18, *I* ^*2*^ = 78%, Figure 4A) or cardiac death (HR = 1.09, 95% CI = 0.80–1.48; *p* = .59, *I* ^*2*^ = 80%, Figure 4B). However, PPIs significantly reduced the risk of GI complications (HR = 0.44, 95% CI = 0.30–0.64; *p <* .0001, *I* ^*2*^ = 19%, Figure 5).

**TABLE 3:** Meta-analysis on outcomes.

Outcome	Number of studies	Pooled HR (95%CI)	*P*-value	Heterogeneity		Analysis model
				*P*_h_	*I* ^*2*^ (%)	
MACEs	18	1.15 (1.06–1.26)	0.001	0.0007	59	R
NACEs	4	1.02 (0.93–1.13)	0.67	0.14	45	F
MI	13	1.18 (1.11–1.24)	<0.00001	0.26	18	F
Stroke	3	1.18 (1.03–1.35)	0.02	0.25	28	F
Revascularization	7	1.17 (1.06–1.30)	0.02	0.19	31	F
Stent thrombosis	11	1.21 (1.03–1.42)	0.02	0.65	0	F
All-cause mortality	13	1.15 (0.94–1.41)	0.18	<0.00001	78	R
Cardiac death	5	1.09 (0.80–1.48)	0.59	0.0006	80	R
GI complications	3	0.44 (0.30–0.64)	<0.0001	0.29	19	F

*MACEs*, major adverse cardiovascular events; *NACEs*, net clinical adverse events; *MI*, myocardial infarction; *GI,* gastrointestinal*; HR*, effect estimates of hazard ratio; *CI*, confidence interval; *R*, random effect model; *F*, fixed effect model.

### 3.3 Publication bias

A funnel plot was drawn for the primary outcome, and it showed symmetry on visual inspection, indicating that publication bias was not large (Supplementary Figure S1).

### 3.4 Sensitivity analysis

No single study markedly altered the overall effect in the sensitivity analysis, suggesting that the pooled HR of MACEs was stable.

## 4 Discussion

This systematic review and meta-analysis evaluated the efficacy and safety of the concomitant use of PPIs with aspirin-clopidogrel DAPT in CHD. The results showed that PPI coadministration decreased the risk of GI complications but could increase the rates of MACEs, stroke, revascularization and ST, in line with previous studies (Melloni et al., 2015; Hu et al., 2018). There were also no significant differences in the risks of all-cause mortality and cardiac death (Hu et al., 2018; Li et al., 2021; Melloni et al., 2015).

Interestingly, the incidence of MI in the PPI group was significantly increased in our study, inconsistent with previous meta-analysis results (Hu et al., 2018; Li et al., 2021). Several reasons account for this outcome. On the one hand, PPIs could decrease the effects of aspirin and clopidogrel on platelet aggregation (Zuern et al., 2010). On the other hand, it has been reported that PPIs could augment cardiovascular risk *via* platelet-independent biological pathways. One suggested mechanism is that PPIs inhibit the enzyme activity of dimethylarginine dimethylaminohydrolase (DDAH), thereby blocking the degradation of endothelial asymmetrical dimethylarginine (ADMA), an endogenous and competitive inhibitor of nitric oxide synthase. Excess ADMA in turn leads to impaired endothelial nitric oxide (NO) generation and reduced vascular function (Ghebremariam et al., 2013; Nolde et al., 2021; Zhai et al., 2022). In addition, a study investigating the long-term effect of PPIs on endothelial dysfunction found that chronic exposure to PPIs could expedite endothelial aging, which might explain the increased cardiovascular events (Yepuri et al., 2016). Therefore, the benefits for GI should be weighed against the recurrent ischemic cardiovascular events. Moreover, we found that there was no significant difference in the risk of NACEs between the two groups, although the risk was higher in the PPI group than in the non-PPI group.

Most interestingly, the elevated risk of MACEs for PPI users might be affected by the PPI subtype and population. *In vitro* studies suggested that different types of PPIs could affect CYP2C19 differently. Based on drug-drug interaction studies, the clinically relevant interaction tendency was the greatest for omeprazole and esomeprazole, with a moderate probability for lansoprazole and the lowest for pantoprazole and rabeprazole (Li et al., 2004; Norgard et al., 2009; Valgimigli et al., 2018). These results prompted our subgroup analyses of PPI subclasses. In agreement with the findings by Sherwood (Sherwood et al., 2015), the use of esomeprazole was associated with an increased risk of MACEs. Physicians should consider the potential risks with different PPIs when prescribing them for individual patients taking aspirin and clopidogrel. Because PCI with stent implantation is the most common interventional treatment for patients with coronary disease, we further performed stratification analyses of the population. In patients following coronary stenting, the occurrence of MACEs was higher in PPI users than in non-PPI users, which could have been driven by the significantly increased risk of ST (Zou et al., 2014).

In addition, we found that the length of follow-up time was quite different among our included studies, with a certain impact on the evaluation of MACEs (Harjai et al., 2011; Maret-Ouda et al., 2021). No previous meta-analyses were performed to evaluate such a difference. The incidence of MACEs varied according to different follow-up times in our study. When the follow-up periods were shorter than 12 months, there was no significant difference between the two groups, while the incidence of MACEs in the PPI group was significantly higher than that in the non-PPI group with longer follow-up (≥12 months). Consequently, long-term follow-up seems to be necessary for cardiovascular event investigations. Furthermore, the results were also inconsistent in different types of studies. The data from observational studies revealed that the use of PPIs increased the risk of MACEs, while the limited data from RCTs showed no significant difference.

There were several limitations to this study. First, most of our included articles were observational studies, and selection bias, along with unmeasured confounding, could account for these findings. Although we extracted the adjusted HRs, our results might still be biased by residual confounding. Second, a small number of RCTs (2 eligible for meta-analysis) were included, and the sample size of some subgroups might have been too small to indicate statistical significance and limit the representativeness of the results, again prompting more RCTs to assess the clinically relevant interactions. Third, we excluded many studies due to the inability to extract data, resulting in some bias. Fourth, subgroup analysis was conducted according to different PPI subtypes, populations, follow-up times and study types to analyze the heterogeneity in our study; however, clinical details, including the duration of DAPT and PPIs, type of stent, CYP2C19 genotypes, and concomitant diseases (such as diabetes), were insufficient in some articles, also potentially leading to heterogeneity among studies. Moreover, the included literature did not stratify the participants by the risk of cardiovascular events, and GI bleeding limited the evaluation of clinical outcomes. Thus, further studies regarding the efficacy and safety of concomitant use of PPIs with aspirin-clopidogrel DAPT should consider these limitations.

## 5 Conclusion

The concomitant use of PPIs was associated with a reduced risk of GI complications, while it could increase the rates of MACEs (particularly in patients receiving esomeprazole or with coronary stenting), MI, stroke, revascularization, and ST in CHD patients receiving aspirin-clopidogrel DAPT. There was no clear evidence of associations between PPI use and NACEs, all-cause mortality, or cardiac death. These results could have been affected by the follow-up time and study type. In light of the limitations of the current systematic review and meta-analysis, large-scale RCTs with longer-term follow-up are warranted to evaluate the safety and efficacy of PPIs with DAPT.

## Data Availability

The original contributions presented in the study are included in the article/Supplementary Material, further inquiries can be directed to the corresponding author.
