# Supplementary material for: Efficacy and safety of concomitant use of proton pump inhibitors with aspirin-clopidogrel dual antiplatelet therapy in coronary heart disease: A systematic review and meta-analysis
Source: Front Pharmacol. 2023 Jan 10;13:1021584. doi: 10.3389/fphar.2022.1021584 (PMC9871580; doi:10.3389/fphar.2022.1021584)
Supplement: Supplementary file 1 [file DataSheet1.ZIP › Supplementary Table S1.docx]

**Supplementary Table S1 |** Quality evaluation of RCTs included according to Jadad Score

| **Study** | **Randomization present** | **Allocation concealment** | **Blinding** | **Follow up** | **Total score** |
| --- | --- | --- | --- | --- | --- |
| Gargiulo 2016 | 1 | 1 | 1 | 1 | 4 |
| Bhatt 2010 | 2 | 1 | 2 | 1 | 6 |
| Total possible scores: 0 to 7 points, considered poor quality if < 4. | | | | | |
