# Supplementary material for: Efficacy and safety of concomitant use of proton pump inhibitors with aspirin-clopidogrel dual antiplatelet therapy in coronary heart disease: A systematic review and meta-analysis
Source: Front Pharmacol. 2023 Jan 10;13:1021584. doi: 10.3389/fphar.2022.1021584 (PMC9871580; doi:10.3389/fphar.2022.1021584)
Supplement: Supplementary file 1 [file DataSheet1.ZIP › Supplementary Table S2.docx]

**Supplementary Table S2 |** Quality evaluation of observational studies included according to the NOS

| **Study** | **Selection** | | | | **Comparability** | **Outcome** | | | **Score** |
| --- | --- | --- | --- | --- | --- | --- | --- | --- | --- |
|  | **A** | **B** | **C** | **D** | **E** | **F** | **G** | **H** |  |
| Mohammed 2021 | ★ | ★ | ★ | ★ | ★★ |  | ★ |  | 7 |
| Maret-Ouda 2021 | ★ | ★ | ★ | ★ | ★★ |  | ★ | ★ | 8 |
| Zhu 2017 | ★ | ★ | ★ | ★ | ★★ | ★ | ★ | ★ | 9 |
| Chandrasekhar 2016 | ★ | ★ | ★ | ★ | ★★ | ★ | ★ | ★ | 9 |
| Weisz 2015 | ★ | ★ | ★ | ★ | ★★ | ★ | ★ | ★ | 9 |
| Zou 2014 | ★ | ★ | ★ | ★ | ★★ | ★ | ★ | ★ | 9 |
| Hokimoto 2014 | ★ | ★ | ★ | ★ | ★★ |  | ★ | ★ | 8 |
| Goodman 2012 | ★ | ★ | ★ | ★ | ★★ | ★ | ★ | ★ | 9 |
| Aihara 2012 | ★ | ★ | ★ | ★ | ★★ | ★ | ★ | ★ | 9 |
| Simon 2011 | ★ | ★ | ★ | ★ | ★★ |  | ★ | ★ | 8 |
| Harjai 2011 | ★ | ★ | ★ | ★ | ★ | ★ | ★ |  | 7 |
| Burkard 2011 | ★ | ★ | ★ | ★ | ★ | ★ | ★ | ★ | 8 |
| Gaglia 2010 | ★ | ★ | ★ | ★ |  | ★ | ★ | ★ | 7 |
| Tentzeris 2010 | ★ | ★ | ★ | ★ | ★★ |  | ★ | ★ | 8 |
| Sarafoff 2010 | ★ | ★ | ★ | ★ | ★★ | ★ |  | ★ | 8 |
| O'Donoghue 2009 | ★ | ★ | ★ | ★ | ★★ | ★ | ★ | ★ | 9 |
| Total possible scores: 0 to 9 points, considered poor quality if < 7. | | | | | | | | | |

*A*,Representativeness of the exposure cohort; *B*, Selection of the non-exposed cohort; *C*, Determination of exposure factor; *D*, Outcome indicators not present at the beginning of study; *E*, Control for important confounders or additional factors; *F*, Evaluation of outcome indicators; *G*, The follow-up time is long enough; *H*, Adequacy of follow-up cohorts; ★, one point
