# Supplementary figures and images for: Efficacy and safety of concomitant use of proton pump inhibitors with aspirin-clopidogrel dual antiplatelet therapy in coronary heart disease: A systematic review and meta-analysis
Source: Front Pharmacol. 2023 Jan 10;13:1021584. doi: 10.3389/fphar.2022.1021584 (PMC9871580; doi:10.3389/fphar.2022.1021584)

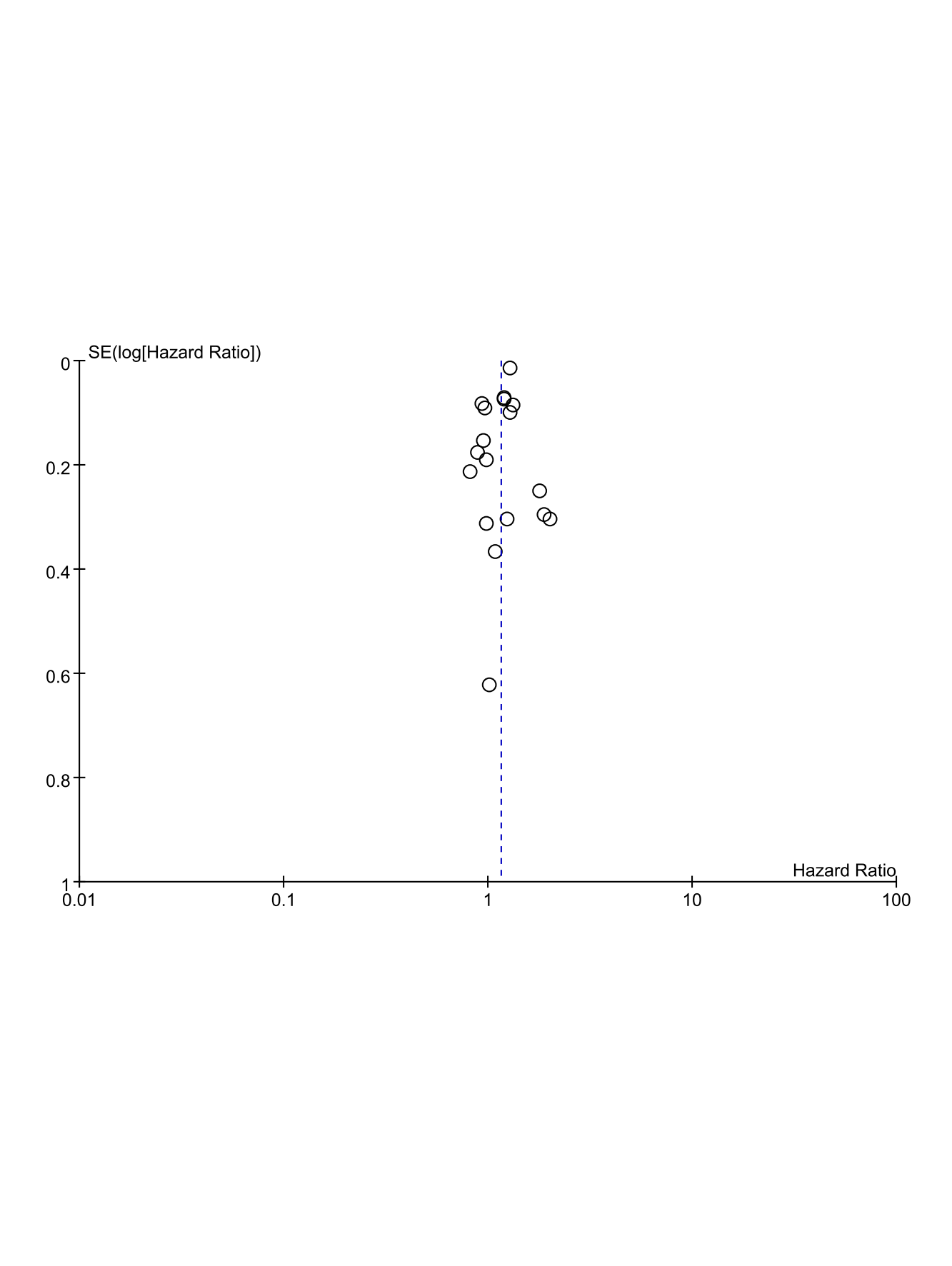

Supplement: Supplementary file 1 [file DataSheet1.ZIP › Supplementary Figure S1.tif]
